# Therapeutic Assessment of TrkB Agonist in a Unilateral Blast-Induced Hearing Loss Mouse Model

**DOI:** 10.3390/audiolres16020036

**Published:** 2026-02-28

**Authors:** Sung Kyun Kim, Han-Gyu Bae, Jun Hee Kim

**Affiliations:** Kresge Hearing Research Institute, Department of Otolaryngology-Head and Neck Surgery, University of Michigan, Ann Arbor, MI 48109, USA; sungkyk@umich.edu (S.K.K.); hangyub@umich.edu (H.-G.B.)

**Keywords:** blast, hearing loss, brain derived neurotrophic factor

## Abstract

Background/Objectives: Blast-induced hearing loss (BIHL) is a major concern, particularly for military personnel, and is linked to impaired auditory neuron survival and synaptic plasticity. This study investigates the potential of the TrkB agonist 7,8-dihydroxyflavone (7,8-DHF) to reduce the severity of BIHL and promote recovery in a mouse model. Methods: Eight-week-old male C57BL/6J mice were used. A custom-built, compressed air-driven system utilizing a modified paintball apparatus was employed to deliver controlled unilateral double blasts (~22 psi exposure pressure) to the left ear. The blasts were administered 30 min apart. Immediately following the second blast, mice received either 7,8-DHF (10 mg/kg) or vehicle (10% DMSO) via intraperitoneal injection. Auditory brainstem responses (ABRs) were measured in both ears at baseline (pre-blast) and at several post-exposure time points. Results: The consecutive blast exposure induced a significant elevation in ABR thresholds, indicative of hearing loss, in both the ipsilateral (exposed) and contralateral (unexposed) ears of vehicle-treated mice. Notably, mice treated with 7,8-DHF demonstrated a marked improvement in hearing recovery compared to the vehicle group. Significant reductions in ABR thresholds were observed in the ipsilateral ear at 4 weeks post-blast (*p* < 0.0001) and in the contralateral ear as early as 1-week post-blast (*p* = 0.0236). However, the recovery was partial, with ABR thresholds plateauing after 4 weeks. Conclusions: A controlled blast model demonstrates that systemic administration of the TrkB agonist 7,8-DHF exerts a protective effect, partially restoring auditory function after blast injury. This supports the therapeutic potential of targeting the BDNF-TrkB signaling pathway for managing BIHL.

## 1. Introduction

Hearing loss is a one of the most common service-connected disabilities among U.S. veterans, and a substantial proportion of cases is associated with noise and blast exposure during military service. The U.S. Department of Veterans Affairs (VA) reports that hearing loss is one of the most prevalent service-connected disabilities, affecting over 4.7 million veterans in 2024 [1]. It is expected that most of the cases are caused by loud noise exposure such as gun firing and explosion. Indeed, a study on U.S. Army soldiers deployed to Iraq and Afghanistan showed that approximately 15–20% of them reported symptoms of blast-induced hearing loss (BIHL) [2,3]. This type of hearing loss is not only problem for veterans, but also for individuals who work in environments with blast overpressure events (e.g., military explosions, industrial blasts) and high-level impact or impulse noises (e.g., pile drivers, industrial impact tools), such as certain construction and industrial settings. Military personnel are frequently exposed to extremely high-intensity impulse and blast noises during training and combat. Reported peak levels typically range from about 140 dB to over 190 dB peak (dBP), substantially exceeding the 140 dBP limit generally recognized as the maximum safe level for a single unprotected impulse exposure. Small arms and rifles commonly produce peaks around 155–160 dB at the shooter’s ear, many heavier weapon systems reach 160–180 dB, and in extreme testing or demolition scenarios, impulse noise can approach or exceed 190–195 dB near the source [4,5]. These levels provide important context for the blast intensities used in the present experimental model.

Despite its prevalence, progress toward targeted therapies has been slowed by variability across injury models and incomplete alignment with real-world exposure scenarios. Preclinical blast systems typically rely on large shock tubes or explosives that are costly, logistically complex, and often deliver bilateral, diffuse exposure. To improve accessibility and experimental control, we adopted a custom compressed-air apparatus based on a modified paintball marker that generates calibrated, repeatable overpressure pulses. The system allows precise control of waveform parameters and incorporates head positioning and shielding to expose a single ear, enabling unilateral injury [6,7,8,9]. This design captures common asymmetric blast geometries encountered operationally, improves within-subject comparisons, and lowers the barrier to adoption by reducing cost and complexity while maintaining experimental rigor.

Exposure of the blast leads to both temporary and permanent hearing loss, tinnitus, and other auditory processing disorders [10,11], but the mechanisms underlying BIHL are complex. Brain-derived neurotrophic factor (BDNF) and its receptor, tropomyosin receptor kinase B (TrkB) signaling is a compelling mechanistic and therapeutic axis for acoustic and blast trauma. BDNF plays a critical role in neuronal survival, plasticity, and synaptic transmission within the auditory system [12,13]. Studies have shown that BDNF-TrkB signaling is essential for the development and maintenance of cochlear hair cells and spiral ganglion neurons, which are particularly vulnerable to blast-induced damage [12,14,15,16,17,18]. 7,8-Dihydroxyflavone (7,8-DHF) is a small molecule TrkB agonist that readily crosses the blood–brain barrier (BBB) and exerts neuroprotective effects in various neurological disorders, including noise-induced synaptopathy [19,20,21,22].

Together, these previous studies motivate and support testing TrkB activation as an acute post-exposure intervention for BIHL. Here, we examined whether 7,8-DHF can attenuate BIHL and promotes hearing recovery, as assessed by ABR thresholds, potentially through engagement of BDNF–TrkB signaling.

## 2. Materials and Methods

### 2.1. Animals

Eight-week-old C57BL/6J mice of both sexes were purchased from Jackson Lab and used in this study. The required animal number for experiment was decided using sample size calculator (Arifin WN. Sample size calculator. Available from: http://wnarifin.github.io, accessed on 25 February 2026). Mice were randomly allocated into experimental groups and housed in each cage with a 12-h light/dark cycle and ad libitum food/water access. All experimental procedures were approved by the Institutional Animal Care and Use Committee (IACUC) of the University of Michigan (Approval Number: PRO00011243) and were conducted in accordance with the National Institutes of Health guidelines for the care and use of laboratory animals.

### 2.2. Drug and Experimental Protocol

**Figure 1:**
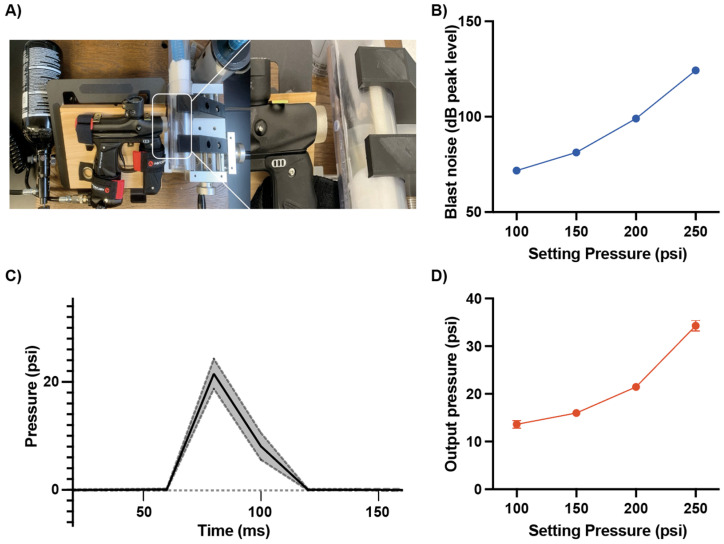
Noise level and pressure output from the custom compressed air blast device. (**A**) Photo of the modified compressed air blast device and the exposure interface used to deliver a controlled unilateral blast to the experimental subject. (**B**) Calibration curve showing the relationship between regulator setting pressure (psi) and peak blast noise level (dB peak level), demonstrating increased acoustic output with higher setting pressure. Blast noise levels (dB SPL peak level) were recorded with a calibrated microphone at the exposure position for single blasts delivered at 100–250 psi. (**C**) Representative pressure–time waveform of the blast overpressure pulse (psi) recorded at the exposure port, illustrating the rapid rise and subsequent decay of the pressure transient. The plot was presented with averaged pressure level from 20 measurement with solid line and s.d. with dashed line). A 200-psi setting produced ~22 psi. (**D**) Relationship between regulator setting pressure and peak output overpressure. A fast-response pressure transducer at the exposure position measured peak overpressure, which scaled with setting pressure. Data are mean ± SD of repeated blasts.

Mice were exposed to the unilateral double blast exposures on the left side. A 200 psi setting pressure results in ~22 psi exposure pressure (Figure 1). ABRs in both ears were measured at baseline and at multiple post-exposure time points (at 3 h, 1 day, 1, 4, and 8 weeks). Three experimental groups were included: (1) Sham (vehicle injection without blast), (2) Blast with vehicle, and (3) Blast with 7,8-DHF. In the blast-exposed groups, ABRs were recorded at baseline (before blast) and at the specified post-blast time points. In the non-blast groups, ABRs were recorded at time-matched intervals following injection. 7,8-DHF was purchased from Sigma-Aldrich (D5446, St. Louis, MO, USA). All animals received either 10 mg/kg of 7,8-DHF [23,24] or vehicle (10% DMSO in PBS) via intra-peritoneal (i.p.) injection within 30 min post blast exposure.

### 2.3. Blast Generating System

A portable blast system was established using paintball apparatus (Mini GS, Empire Paintball, Sewell, NJ, USA) on securely mounted in a fixed position following a previously published paper [7]. High pressure air was released through the blast device that connected to a compressed air tank (48/3000 HPA tank, Maddog Sports, CA, USA). A plexiglass cylinder was used to hold the mouse during blast exposure. The cylinder consisted of an inner pipe (36 mm inner diameter) and an outer pipe (42 mm outer diameter), and it was mounted on a manually adjustable two-dimensional positioning stage (Velmex Inc., Bloomfield, NY, USA). A blast was delivered to the mouse through 7 mm hole that was made in the cylinder to expose the external ear, and the cylinder was mounted on an x-y table (Velmex Inc., Bloomfield, NY, USA). The distance from the apparatus outlet to the target on the mouse was kept as 1 cm across all experiments.

Before blast exposure, mice were anesthetized with isoflurane (2.5% isoflurane with 1 L/min O_2_ flow rate), maintaining anesthesia by flowing isoflurane through tubing connected into the inside of the cylinder. To minimize physical damage from high pressure, sponge foam was strategically placed within the cylinder as a cushion for the head.

### 2.4. Measurement of Output Pressure and Sound Intensity

To characterize the blast output, measurements of both output pressure and sound intensity were performed across a range of pressure settings (100 psi to 250 psi). Output pressure was measured using a pressure transducer (Honeywell, Morristown, NJ, USA) positioned at the location where the targeted region of the left ear would be placed during experiments. The pressure transducer was set to record pressure readings every 20 ms to capture the dynamic changes in pressure during the blast. The voltage reading from the pressure transducer was back calculated to psi using Arduino chip board (Arduino SA, Ivrea, Italy). By reading baseline as 14.5038 psi as 1 bar of atmosphere, additional pressure was calculated by subtracting the reading values with baseline. Based on these calibrations, the setting pressures used in this study (100–250 psi) corresponded to sound pressure levels of approximately 70–125 dB peak SPL at the mouse ear, as shown in Figure 1.

Sound intensity measurements were conducted using a HT-80A sound level meter (Xintai Instrument Co., Dongguan City, Guangdong, China) positioned similarly to the pressure transducer. The decibel meter recorded the peak sound intensity generated by the blast at each pressure setting, providing a measure of the acoustic energy delivered to the animal.

### 2.5. Auditory Brainstem Response

Auditory brainstem responses (ABRs) were recorded to assess hearing thresholds. Mice were anesthetized with 3.0% isoflurane (induction) and maintained at 2.5% (1 L/min O_2_). ABR recordings were performed inside a sound-attenuating chamber (Med Associates, Albans, VT, USA) with subdermal electrodes (Rochester Electro-Medical, Lutz, FL, USA) placed at the vertex (active), ipsilateral mastoid (reference), and contralateral mastoid (ground). Acoustic stimuli were generated by an Auditory Evoked Potentials Workstation (Tucker-Davis Technologies, Alachua, FL, USA). Clicks (0.1 ms duration, amplitude-modulated square waves) were delivered to each ear via a closed-field system using a 10 cm plastic tube (Tygon; 3.2 mm outer diameter) connected to TDT Multi-Field Magnetic Speakers. The speaker tube was alternately inserted into the left and right external ear canal, and thresholds were measured separately for each ear. Click stimuli intensities ranged from 90 to 10 dB SPL (5 dB decrements), with 512 recordings averaged (16 Hz sampling rate). Tone stimuli (4, 8, 12, 16, and 32 kHz) were presented at intensities ranging from 90 to 20 dB SPL (10 dB decrements), with the same sampling rate. The threshold was defined as the lowest intensity at which a consistent, reproducible ABR wave I-III was observed.

### 2.6. Data Analysis and Statistical Analysis

Data acquisition and analysis was performed blindly, and group allocation was revealed at the end of analysis to minimize any bias. Experimental data were processed without any exclusion and visualized using Prism software v9 (GraphPad Software, San Diego, CA, USA). To test statistical significance between groups, two-way ANOVA test with multiple comparison was conducted, and the *p* values were adjusted with Bonferroni correction. Data are expressed as mean ± standard mean error (SEM) or standard deviation (SD). *p* < 0.05 was considered as statistically significant.

## 3. Results

### 3.1. Establishment and Calibration of the Custom Compressed Air Blast Device Utilizing the Modified Paintball Apparatus

To establish and validate the blast injury mouse model, we first characterized the output of our compressed air-driven blast-generating device. Output pressure and sound intensity were measured across a range of the setting pressure from the compressed air tank (100–250 psi) using a pressure transducer and a decibel meter, respectively. Both noise level and output pressure increased proportionally with the setting pressure (Figure 1). Noise levels ranged from 71.81 ± 0.66 dB (10 times of measurements) at the lowest setting pressure (100 psi) to 124.31 ± 3.13 dB (10 times of measurements) at the highest setting pressure (250 psi, Figure 1A). Output pressure increased as the setting pressure increased. Output pressure varied from 13.61 ± 3.63 psi (20 times of measurements) at 100 psi to 34.30 ± 5.05 psi (20 times of measurements) at 250 psi (Figure 1B). These calibrations guided selection of the unilateral exposure parameters used in the study.

### 3.2. Effects of Single or Double Blast Exposure on Hearing Function

**Figure 2:**
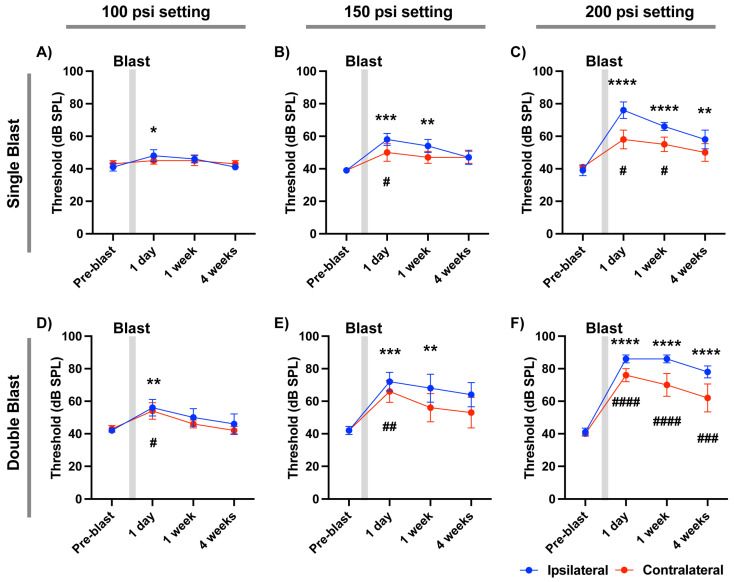
ABR threshold changes responding to click stimuli following blast exposure across regulator settings. (**A**–**F**) ABR thresholds were measured in the ipsilateral (blue) and contralateral (red) ears at baseline (pre-blast), 1 day, 1 week, and 4 weeks post-exposure. The gray bar indicates the blast time. ABR thresholds in mice given single blast exposure at various pressure settings at 100, 150, and 200 psi (**A**,**B**). Double-blast exposure (two pulses, 10 min interval) at 100, 150, or 200 psi (**D**–**F**). Data are shown with mean ± SEM. Asterisks (ipsilateral) and pound (contralateral) indicate a significant change vs. the corresponding pre-blast value within the same ear. Statistical significances were acquired by two-way repeated-measures ANOVA tests with multiple comparison with Bonferroni correction. *, #, *p* < 0.05; **, ##, *p* < 0.005; ***, ###, *p* < 0.001; ****, ####, *p* < 0.0001.

We next examined ABR thresholds in mice exposed to a single blast at varying tank pressures (100, 150, and 200 psi). ABR thresholds for both ears were measured pre-blast and at 1 day, 1 week, and 4 weeks post-blast (Figure 2A–C). Even the lowest setting (100 psi; ~14 psi output) caused a significant increase in ABR threshold from the ipsilateral ear at 1 day post-blast (*p* = 0.0273, N = 5; Bonferroni correction), with no significant change in the contralateral ear (*p* > 0.9999). Thresholds recovered to baseline by 7 days (*p* = 0.1617). At the highest setting pressure (200 psi; ~22 psi output), the ipsilateral ear exhibited a marked threshold elevation at 1 day post-blast (*p* < 0.0001, N = 5 mice, multiple comparison with Bonferroni correction), which only partially recovered by 4 weeks post-blast (*p* = 0.0044). The contralateral ear also showed a mild but significant threshold shift at 1 day post-blast (*p* = 0.0112), which mostly recovered to baseline by 4 weeks (*p* = 0.3057). These results demonstrate that a single blast exposure in this system can induce both temporary and persistent hearing impairment.

To assess the effect of blast exposure frequency, mice were exposed to two blasts 10 min apart. At 100 psi, double blasts significantly elevated ABR thresholds in both ipsilateral and contralateral ears at 1 day post-blast (ipsilateral: *p* = 0.0081; contralateral: *p* = 0.0444; N = 5; Bonferroni correction). Thresholds fully recovered by 4 weeks post-blast (ipsilateral: *p* = 0.2044; contralateral: *p* > 0.9999). At 200 psi, double blasts induced severe threshold shifts in both ears (*p* < 0.0001 for all comparisons), which persisted through 4 weeks post-injury (Figure 2D–F). This condition produced the most pronounced and sustained hearing deficits observed in our experiments.

### 3.3. Frequency-Specific ABR Threshold Elevations After Unilateral Blast

**Figure 3:**
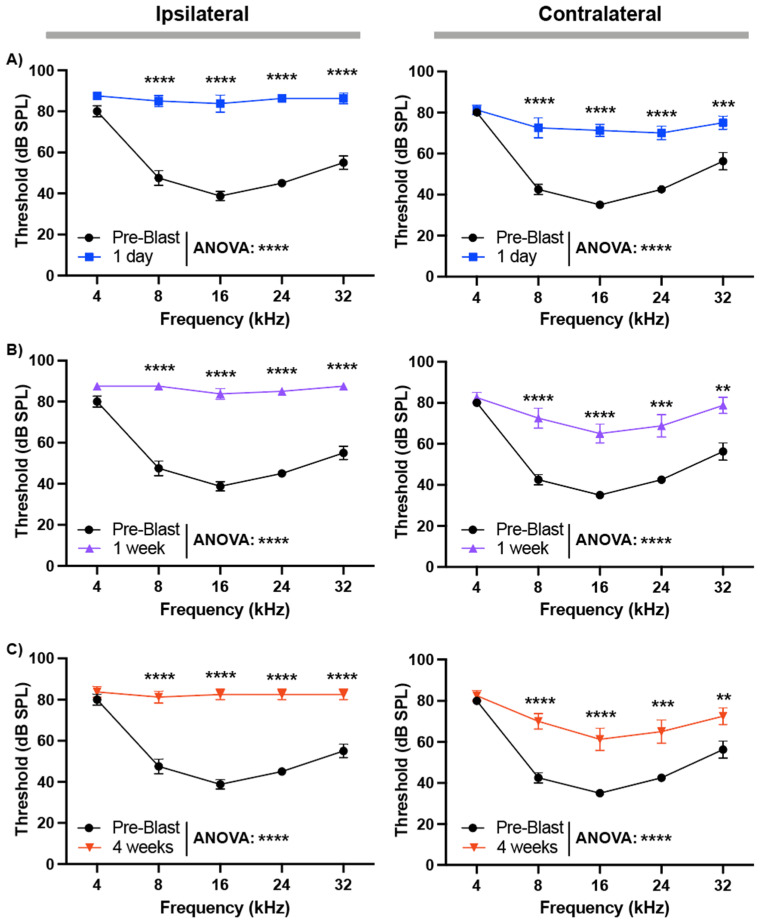
ABR threshold changes in response to pure tone stimulation after double blast exposure. Tone ABR thresholds were measured at 4–32 kHz in the ipsilateral (left column) and contralateral (right column) ears before exposure (pre-blast, black) and at (**A**) 1 day (blue), (**B**) 1 week (violet), and (**C**) 4 weeks (red) after unilateral blast. Across both ipsilateral and contralateral sides, blast produced robust, broadband threshold elevations that were greatest acutely and remained elevated through 4 weeks, with larger deficits ipsilaterally. Data are presented as mean ± SEM. Statistical significances were acquired from two-way ANOVA test with multiple comparison after Bonferroni correction. **, *p* < 0.005; ***, *p* < 0.001; ****, *p* < 0.0001.

We further evaluated frequency-specific hearing loss after double blast exposure at 200 psi. ABR thresholds were measured in response to pure tones from 4 kHz to 32 kHz. In the ipsilateral ear, thresholds were significantly elevated at all tested frequencies 1 day post-blast (*p* < 0.0001, N = 8; two-way ANOVA) and remained significantly elevated at 4 weeks (*p* < 0.0001; Figure 3A–C). The contralateral ear also showed significant threshold elevations at 1 day (*p* < 0.0001), with partial but incomplete recovery by 4 weeks (*p* < 0.0001; Figure 3A–C). Collectively, these data demonstrate that higher-intensity and repeated blast exposures cause more severe and persistent hearing loss, with greater impact on the ipsilateral ear but measurable effects bilaterally.

### 3.4. Therapeutic Effect of 7,8-DHF on Hearing Loss Following Repeated Blast Exposure

We investigated whether pharmacological activation of BDNF/TrkB signaling with 7,8-dihydroxyflavone (7,8-DHF) could mitigate blast-induced hearing impairment. To induce persistent auditory deficits, mice were subjected to double blast exposures (200 psi setting pressure; ~22 psi overpressure, ~127 dB SPL output) separated by a 10-min interval. Immediately after the second blast, 7,8-DHF (10 mg/kg) or vehicle (10% DMSO) was administered intraperitoneally. ABR thresholds were measured at pre-blast (baseline) and at 3 h, 1 day, 1 week, 4 weeks, and 8 weeks post-blast.

**Figure 4:**
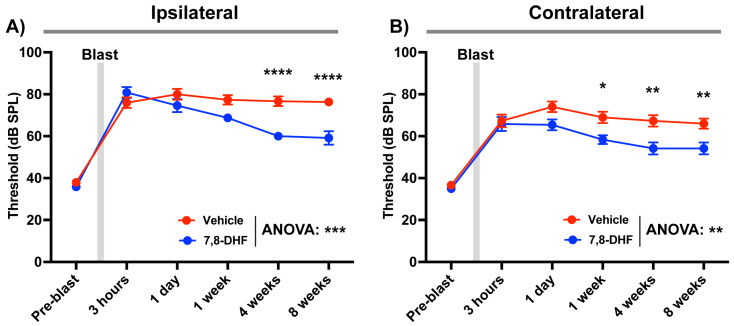
TrkB activation with 7,8-DHF attenuates blast-induced ABR threshold elevations. (**A)** Ipsilateral ear. (**B**) Contralateral ear. Mice received a unilateral double-blast (10 min interval; peak ~22 psi per pulse) and were treated immediately afterward with 7,8-DHF (10 mg/kg, i.p.) or vehicle (10% DMSO). Click-evoked ABR thresholds were measured at pre-blast, 3 h, 1 day, 1 week, 4 weeks, and 8 weeks. 7,8-DHF accelerated recovery and reduced the magnitude of chronic threshold elevation, with a larger effect in the ipsilateral ear. Data are presented as mean ± SEM. Statistical significances were acquired from two-way ANOVA test with multiple comparison after Bonferroni correction. *, *p* < 0.05; **, *p* < 0.005; ***, *p* < 0.001; ****, *p* < 0.0001.

Consistent with our earlier findings, double blast exposure caused significant and sustained ABR threshold elevations in both ipsilateral and contralateral ears in the vehicle-treated group, persisting through 8 weeks (Figure 4). In contrast, 7,8-DHF treatment promoted partial recovery in both ears, with significant improvements detected by two-way ANOVA (ipsilateral: *p* = 0.0002; contralateral: *p* = 0.0021). Multiple comparison analysis with Bonferroni correction revealed that recovery became significant at 4 weeks post-blast in the ipsilateral ear (*p* < 0.0001) and at 1 week in the contralateral ear (*p* = 0.0236). The improvement plateaued by 4 weeks and was maintained through 8 weeks. These results demonstrate that systemic administration of 7,8-DHF after blast injury can partially restore auditory function, supporting BDNF/TrkB pathway activation as a potential therapeutic approach for mitigating long-term blast-induced hearing loss.

## 4. Discussion

This study establishes and characterizes a portable, precisely controllable compressed air system for modeling BIHL in mice. By pairing this platform with ABR measurements, we defined a clear dose–response relationship between blast overpressure and threshold shift using a low-cost apparatus. By varying both overpressure and the number of exposures, the paradigm recapitulates temporary and permanent threshold shifts, providing flexible control over injury severity. We also evaluated a therapeutic pathway by activating BDNF/TrkB signaling with the small-molecule agonist 7,8-DHF, which conferred robust, albeit incomplete, protection against PTS.

A key feature of our approach is unilateral blast exposure, which contrasts with traditional blast research that typically employs frontal exposure paradigm [10,25,26]. In blast exposure from the front using a conventional blast chamber, partial hearing recovery was observed. However, in our unilateral blast model, the ipsilateral ear failed to recover over four weeks, with only the contralateral ear demonstrating partial improvement [15,27]. In real-world blast events, whether in combat field or industrial situations, blast overpressure frequently originates from unexpected directions outside the field of vision. Frontal exposure allows for a visual cue, potentially enabling protective reflexes or mitigating behaviors. By delivering the blast unilaterally, this study more closely mimics the unpredictable nature of real-world blast situations, where individuals have limited opportunity for anticipatory or defensive responses.

An important limitation of the current device is that it reproduces the positive-pressure component of the blast wave but not the negative-pressure (rarefaction) phase present in true explosions [28,29]. This omission reflects the physical design constraints of our air-pressure generator. To overcome this limitation, future studies should incorporate diaphragm-based elements from conventional shock-tube systems to generate a biphasic waveform with more realistic rise times and durations, enabling closer alignment to real-world blast physics.

While treatment with 7,8-DHF led to significant hearing recovery starting at one-week post-treatment, full restoration of hearing to pre-blast level was not achieved, resulting in partial recovery. In this study, our conclusions are explicitly limited to functional protection, as assessed by ABR thresholds, against blast-induced hearing loss. The observed protection could arise from effects at multiple levels of the auditory system, including the middle ear, cochlea, and central auditory pathways. Future work will need to combine functional measures with detailed anatomical and physiological assessments, including cochlear hair cell and synapse counts and middle-ear evaluations to clarify the underlying BDNF–TrkB signaling mechanisms. Several factors may contribute to this incomplete recovery. One possibility is that the systemic 7,8-DHF injection may not have resulted in sufficient intracochlear drug concentrations. Although 7,8-DHF is blood–brain barrier-permeable, penetration across the blood–labyrinth barrier (BLB) may be suboptimal, limiting intracochlear drug levels after systemic administration [30,31,32]. In addition, it can be presumed that systemic injection may lead to lower drug concentration in the intracochlear distribution [33,34,35]. These considerations support testing local routes (e.g., intratympanic injection) to bypass the blood–labyrinth barrier and achieve higher cochlear exposure [36,37]. Future work should also evaluate dosing paradigms, treatment windows, and combination strategies that optimize TrkB pathway engagement.

We explored additional dosing strategies in preliminary experiments, but their effects appeared limited. Administration of 7,8-DHF at 5 mg/kg (i.p.) did not improve hearing thresholds, and consecutive injections for 3 days after blast exposure likewise showed no detectable effect. We also compared pre- versus post-treatment paradigms of 7,8-DHF administration but did not observe further enhancement of hearing rescue beyond the effects reported in this study. Because we did not conduct a systematic, classical dose–response or timing study, these preliminary observations should be interpreted as hypothesis-generating rather than conclusive. We therefore acknowledge as a limitation that the optimal dose, dosing schedule, and treatment window remain to be defined. Future studies will need comprehensive testing across multiple doses and administration timelines to establish an optimal regimen for blast-induced auditory injury.

Taken together, this study provides a valuable and versatile model for studying BIHL, highlighting the importance of the TrkB signaling pathway in hearing recovery and underscoring the need for further investigation into effective therapeutic strategies for hearing loss following blast exposure.

## 5. Conclusions

A controlled blast model demonstrates that systemic administration of the TrkB agonist 7,8-DHF exerts a protective effect, partially restoring auditory function after blast injury. This supports the therapeutic potential of targeting the BDNF–TrkB signaling pathway for managing BIHL.

## Data Availability

The original contributions presented in this study are included in the article. Further inquiries can be directed to the corresponding author.

## Abbreviations

The following abbreviations are used in this manuscript:

BIHL	Blast-induced hearing loss
7,8-DHF	7,8-dihydroxyflavone
ABRs	Auditory brainstem responses
VA	The U.S. Department of Veterans Affairs
BDNF	Brain-derived neurotrophic factor
TrkB	Tropomyosin receptor kinase B
BBB	Blood-brain barrier
IACUC	Institutional Animal Care and Use Committee
BLB	Blood-labyrinth barrier

## References

[1] Affairs USDoV (2025). Annual Benefits Report.

[2] Reavis K.M., Snowden J.M., Henry J.A., Gallun F.J., Lewis M.S., Carlson K.F. (2021). Blast Exposure and Self-Reported Hearing Difficulty in Service Members and Veterans Who Have Normal Pure-Tone Hearing Sensitivity: The Mediating Role of Posttraumatic Stress Disorder. J. Speech Lang. Hear. Res..

[3] Swan A., Nelson J., Swiger B., Jaramillo C., Eapen B., Packer M., Pugh M. (2017). Prevalence of hearing loss and tinnitus in Iraq and Afghanistan Veterans: A Chronic Effects of Neurotrauma Consortium study. Hear. Res..

[4] Headquarters DotA (2015). Army Hearing Program.

[5] Jokel C., Yankaskas K., Robinette M.B. (2019). Noise of military weapons, ground vehicles, planes and ships. J. Acoust. Soc. Am..

[6] Hines-Beard J., Marchetta J., Gordon S., Chaum E., Geisert E.E., Rex T.S. (2012). A mouse model of ocular blast injury that induces closed globe anterior and posterior pole damage. Exp. Eye Res..

[7] Heldt S.A., Elberger A.J., Deng Y., Guley N.H., Del Mar N., Rogers J., Choi G.W., Ferrell J., Rex T.S., Honig M.G. (2014). A novel closed-head model of mild traumatic brain injury caused by primary overpressure blast to the cranium produces sustained emotional deficits in mice. Front. Neurol..

[8] Guley N.H., Rogers J.T., Del Mar N.A., Deng Y., Islam R.M., D’SUrney L., Ferrell J., Deng B., Hines-Beard J.B., Bu W. (2016). A Novel Closed-Head Model of Mild Traumatic Brain Injury Using Focal Primary Overpressure Blast to the Cranium in Mice. J. Neurotrauma.

[9] Allen R.S., Motz C.T., Singh A., Feola A., Hutson L., Douglass A., Rao S.R., Skelton L.A., Cardelle L., Bales K.L. (2021). Dependence of visual and cognitive outcomes on animal holder configuration in a rodent model of blast overpressure exposure. Vis. Res..

[10] Ouyang J., Pace E., Lepczyk L., Kaufman M., Zhang J., Perrine S.A., Zhang J. (2017). Blast-Induced Tinnitus and Elevated Central Auditory and Limbic Activity in Rats: A Manganese-Enhanced MRI and Behavioral Study. Sci. Rep..

[11] Wang Y., Urioste R.T., Wei Y., Wilder D.M., Arun P., Sajja V., Gist I.D., Fitzgerald T.S., Chang W., Kelley M.W. (2020). Blast-induced hearing impairment in rats is associated with structural and molecular changes of the inner ear. Sci. Rep..

[12] Li N., Chen B., Jia G., Xu R., Xia Y., Lai C., Li G., Li W., Han Y. (2023). Reduced BDNF expression in the auditory cortex contributed to neonatal pain-induced hearing impairment and dendritic pruning deficiency in mice. Reg. Anesth. Pain Med..

[13] Pisani A., Rolesi R., Mohamed-Hizam V., Montuoro R., Paludetti G., Giorgio C., Cocchiaro P., Brandolini L., Detta N., Sirico A. (2025). Early transtympanic administration of rhBDNF exerts a multifaceted neuroprotective effect against cisplatin-induced hearing loss. Br. J. Pharmacol..

[14] Sly D.J., Hampson A.J., Minter R.L., Heffer L.F., Li J., Millard R.E., Winata L., Niasari A., O’lEary S.J. (2012). Brain-derived neurotrophic factor modulates auditory function in the hearing cochlea. J. Assoc. Res. Otolaryngol..

[15] Cho S.-I., Gao S.S., Xia A., Wang R., Salles F.T., Raphael P.D., Abaya H., Wachtel J., Baek J., Jacobs D. (2013). Mechanisms of hearing loss after blast injury to the ear. PLoS ONE.

[16] Niwa K., Mizutari K., Matsui T., Kurioka T., Matsunobu T., Kawauchi S., Satoh Y., Sato S., Shiotani A., Kobayashi Y. (2016). Pathophysiology of the inner ear after blast injury caused by laser-induced shock wave. Sci. Rep..

[17] Johnson Chacko L., Blumer M.J.F., Pechriggl E., Rask-Andersen H., Dietl W., Haim A., Fritsch H., Glueckert R., Dudas J., Schrott-Fischer A. (2017). Role of BDNF and neurotrophic receptors in human inner ear development. Cell Tissue Res..

[18] de Vries I., Schmitt H., Lenarz T., Prenzler N., Alvi S., Staecker H., Durisin M., Warnecke A. (2019). Detection of BDNF-Related Proteins in Human Perilymph in Patients With Hearing Loss. Front. Neurosci..

[19] Akhtar A., Dhaliwal J., Sah S.P. (2021). 7,8-Dihydroxyflavone improves cognitive functions in ICV-STZ rat model of sporadic Alzheimer’s disease by reversing oxidative stress, mitochondrial dysfunction, and insulin resistance. Psychopharmacology.

[20] Fernandez K.A., Watabe T., Tong M., Meng X., Tani K., Kujawa S.G., Edge A.S. (2021). Trk agonist drugs rescue noise-induced hidden hearing loss. JCI Insight.

[21] Xu M., Xia L., Li J., Du Y., Dong Z. (2024). 7,8-Dihydroxyflavone ameliorates cognitive impairment induced by repeated neonatal sevoflurane exposures in mice through increasing tau O-GlcNAcylation. Neurosci. Lett..

[22] Sun S.R., Zhao J.N., Bi P.W., Zhang H.Y., Li G.X., Yan J.Z., Li Y.F., Yin Y.Y., Cheng H. (2025). Pharmacologically activating BDNF/TrkB signaling exerted rapid-acting antidepressant-like effects through improving synaptic plasticity and neuroinflammation. Metab. Brain Dis..

[23] Ren Q., Zhang J.-C., Ma M., Fujita Y., Wu J., Hashimoto K. (2014). 7,8-Dihydroxyflavone, a TrkB agonist, attenuates behavioral abnormalities and neurotoxicity in mice after administration of methamphetamine. Psychopharmacology.

[24] Jhan K.-Y., Chen K.-Y., Chang P.-K., Chiu C.-H., Chou C.-J., Wang L.-C. (2025). 7,8-Dihydroxyflavone provides neuroprotection and rescues behavioral deficits in Angiostrongylus cantonensis-infected mice by ameliorating synaptic loss. J. Microbiol. Immunol. Infect..

[25] Liang J., Yokell Z.A., Nakmaili D.U., Gan R.Z., Lu H. (2017). The effect of blast overpressure on the mechanical properties of a chinchilla tympanic membrane. Hear. Res..

[26] Ewert D.L., Lu J., Li W., Du X., Floyd R., Kopke R. (2012). Antioxidant treatment reduces blast-induced cochlear damage and hearing loss. Hear. Res..

[27] Mao B., Wang Y., Balasubramanian T., Urioste R., Wafa T., Fitzgerald T.S., Haraczy S.J., Edwards-Hollingsworth K., Sayyid Z.N., Wilder D. (2021). Assessment of auditory and vestibular damage in a mouse model after single and triple blast exposures. Hear. Res..

[28] Mao J.C., Pace E., Pierozynski P., Kou Z., Shen Y., VandeVord P., Haacke E.M., Zhang X., Zhang J. (2012). Blast-induced tinnitus and hearing loss in rats: Behavioral and imaging assays. J. Neurotrauma.

[29] Jiang S., Sanders S., Gan R.Z. (2023). Mitigation of Hearing Damage With Liraglutide Treatment in Chinchillas After Repeated Blast Exposures at Mild-TBI. Mil. Med..

[30] Nyberg S., Abbott N.J., Shi X., Steyger P.S., Dabdoub A. (2019). Delivery of therapeutics to the inner ear: The challenge of the blood-labyrinth barrier. Sci. Transl. Med..

[31] Delaney D.S., Liew L.J., Lye J., Atlas M.D., Wong E.Y.M. (2023). Overcoming barriers: A review on innovations in drug delivery to the middle and inner ear. Front. Pharmacol..

[32] Yi Z., Wang X., Yin G., Sun Y. (2025). The Blood-Labyrinth Barrier: Non-Invasive Delivery Strategies for Inner Ear Drug Delivery. Pharmaceutics.

[33] Liu S.S., Yang R. (2022). Inner Ear Drug Delivery for Sensorineural Hearing Loss: Current Challenges and Opportunities. Front. Neurosci..

[34] Szeto B., Chiang H., Valentini C., Yu M., Kysar J.W., Lalwani A.K. (2020). Inner ear delivery: Challenges and opportunities. Laryngoscope Investig. Otolaryngol..

[35] Li Y., Kanzaki S., Shibata S., Nakamura M., Ozaki M., Okano H., Ogawa K. (2020). Comparison of inner ear drug availability of combined treatment with systemic or local drug injections alone. Neurosci. Res..

[36] Xu Y., Bei Z., Li M., Qiu K., Ren J., Chu B., Zhao Y., Qian Z. (2024). Biomaterials for non-invasive trans-tympanic drug delivery: Requirements, recent advances and perspectives. J. Mater. Chem. B.

[37] Hu Y., Fang L., Zhang H., Zheng S., Liao M., Cui Q., Wei H., Wu D., Cheng H., Qi Y. (2023). Emerging biotechnologies and biomedical engineering technologies for hearing reconstruction. Smart Med..

